# Treatment of motor and behavioural symptoms in three Lesch-Nyhan patients with intrathecal baclofen

**DOI:** 10.1186/s13023-014-0208-3

**Published:** 2014-12-12

**Authors:** Marco Pozzi, Luigi Piccinini, Maurizio Gallo, Francesco Motta, Sonia Radice, Emilio Clementi

**Affiliations:** Scientific Institute IRCCS Eugenio Medea, 23842 Bosisio Parini, Lecco, Italy; Scuola di Specializzazione in Medicina Fisica e Riabilitativa, Università di Milano, 20122 Milan, Italy; Paediatric Orthopedics and Traumatology, Children’s Hospital V. Buzzi, 20126 Milan, Italy; Unit of Clinical Pharmacology, Department of Biomedical and Clinical Sciences L. Sacco, “Luigi Sacco” University Hospital, Università di Milano, 20157 Milan, Italy; Unit of Clinical Pharmacology, CNR Institute of Neuroscience, Department of Biomedical and Clinical Sciences L. Sacco, “Luigi Sacco” University Hospital, Università di Milano, Via GB Grassi 74, 20157 Milan, Italy

**Keywords:** Lesch-Nyhan syndrome, Baclofen, Dystonia, Self-injurious behaviour

## Abstract

Current therapies for the Lesch-Nyhan Syndrome (OMIM: 300322) are off-label and experimental, often leading to inconsistent outcomes. We here report the effects of an intrathecal baclofen therapy, carried out at the Scientific Institute Eugenio Medea (Lecco, Italy), on three patients who no longer received benefit from previous therapies. This treatment, as expected, ameliorated the motor symptoms and, unexpectedly, it also improved behavioural components. This result may involve a functional interaction between baclofen and dopamine, complemented by an anxiolytic effect. Our observations provide the rationale for the use of intrathecal baclofen administration in the therapy of the Lesch-Nyhan Syndrome.

## Letter to the editor

### Introduction

The Lesch-Nyhan Syndrome [1] (LN) (OMIM: 300322) involves dystonia, ballism, and self-injurious and aggressive behaviours. Although LN is severely disabling, no therapeutic standard can yet be indicated and treatment proceeds on the basis of isolated observations. Many therapies for LN, both pharmacological (antispastic drugs, antipsychotics, anti-parkinsonian drugs, dietary supplements) and cellular (enzyme replacement and stem cell therapies), are currently experimented, with inconsistent results [2]. We report on three patients with a genetic diagnosis of classical LN, who were referred to the Scientific Institute Eugenio Medea (Lecco, Italy) for rehabilitation. They were treated with intrathecal baclofen (ITB) and showed an improvement regarding both dystonia and pathological behaviours.

## Patients and methods

Patient 1 was 19 years old at referral. During motor development, he never achieved head control, did not crawl or walk; instead, he developed bilateral clubfoot and phasic extensor hypertonia of the upper limbs, with dystonia and ballism. His pathological behaviour involved very severe self-injury and involuntary aggression, by punching and biting. The patient constantly wore whole-body restraints in order to contain these exacerbations. Patient 2 was referred at 39 years of age. He never achieved head control, but crawled and walked until 9, when severe dystonia and ballism of the limbs began. He displayed severe finger biting and required permanent finger protection. Patient 3 was 20 years old at referral. He never achieved head control, crawled scantly and never walked. He developed strong retropulsive reactions, with dystonia involving neck and limbs, and ballism of the arms. By punching, he injured himself and attacked others. Patients were weaned off their previous therapies (Table 1) and subsequently implanted with the intrathecal drug delivery device Synchromed II - 20 ml (Medtronic, Minneapolis, MN, USA). Individual ITB dosages were up titrated to achieve a satisfactory effect on dystonia.

**Table 1:** **Patients’ previous pharmacological therapies**

**Patient**	**Remote therapeutic history**			**Previous therapy before ITB placement**		
**1**	Trihexyphenidyl	4 mg x3/day	Started in 2006 Discontinued in 2010	S-adenosyl methionine	400 mg x4/day	Started in 2000
						Initial efficacy, progressively lost
						Discontinued 24/04/2013
**2**	Risperidone	6 mg /day	Started in 1998 Discontinued in 2011	Levetiracetam	250 mg x3/day	Started in 2002
						No efficacy
						Weaned from 03/01/2014, discontinued 20/02/2014
				Clonazepam	1 mg /day	Started in 2002
						Partial efficacy on spasticity
						Weaned from 12/02/2014, discontinued 21/02/2014
				Sertraline	50 mg /day	Started in 2002
						Scarce efficacy
						Weaned from 08/02/2014, discontinued 22/02/2014
				Zopiclone	7.5 mg /day	Started in 2002
						Good efficacy on sleep improvement
						Weaned from 12/02/2014, discontinued 21/02/2014
**3**	Enzyme replacement therapy	Intrathecal infusion of leukocytes (2/month)	Started in 1996 Discontinued in 2010	Gabapentin	400 mg x4/day	Started in May, 2013
						No efficacy
						Weaned from 23/01/2014, discontinued 15/03/2014
		Intrathecal infusion of mesenchymal stem cells	Single administration in 2012			

## Results

Dystonia was controlled with ITB dosages of 270 to 550 μg per day. ITB improved the quality of sleep for all patients, as expected. This happened in the absence of serious adverse reactions; patient 3 only experienced mild drowsiness. In addition, within three days after reaching the stable ITB dosage, ballism was abolished and aggression and self-injurious behaviours ceased, thus allowing the removal of protective restraints (details in Tables 2 and 3). The beneficial effects of ITB therapy at unchanged dosages persisted throughout the follow up period (5 to 16 months).

**Table 2:** **Patients’ symptoms before and after treatment with intrathecal baclofen**

**Patient**		**1**	**2**	**3**
**Before ITB**	**Dystonia:**	22	23	38
	**UDRS total** ^**a**^			
	**Aggression**	Yes	No	Yes
	**Self-injury** ^**b**^ **:**			
	**Mouth/lip biting**	2-3	2	1-2
	**Finger biting**	10	20	3-4
	**Punching**	10	0	2-3
	**Restraint**	Permanent whole body restraint	Permanent finger protection	None
	**Sleep** ^**c**^ **:**			
	**Awakenings**	7-8	5-6	5-6
	**Sleep hours**	2	4	4
**Date of ITB implantation and age (years)**		29/04/2013 – 19	28/02/2014 – 39	19/03/2014 – 20
**ITB dosage and follow-up duration (months)**		380 μg /day – 16	270 μg /day – 6	550 μg /day – 5
**After ITB**	**Dystonia:**	5	6.5	11
	**UDRS total** ^**a**^			
	**Aggression**	No	No	No
	**Self-injury** ^**b**^ **:**			
	**Mouth/lip biting**	0-1	0	0
	**Finger biting**	3	5	0
	**Punching**	0	0	0
	**Restraint**	Occasional, right arm	None	None
	**Sleep** ^**c**^ **:**			
	**Awakenings**	1-2	1	0
	**Sleep hours**	5	6	5
	**Notes**	Improved verbal communication	-	Persistent moderate nausea, daytime drowsiness

Legend: a) Dystonia was scored using the UDRS scale [3]. Detailed scores are available in Table 3. b) Self-injury was scored by counting the daily episodes of different self-injurious behaviours, following interviews with caregivers. c) Quality of sleep was scored counting the number of awakenings per night and the average hours of uninterrupted sleep, following interviews with caregivers.

**Table 3:** **Detailed scores from patients’ UDRS scales**

**Patient**	**Before ITB**	**After ITB**
**1**	Duration factor: 3	Duration factor: 0
	Motor severity factor: eyes and upper face: 1, lower face: 3, jaw and tongue: 2, larynx: 0, neck: 2, shoulder and proximal arm. 2, distal arm and hand (including elbow): 3, pelvis and proximal leg: 3, distal leg and foot (including knee): 2, trunk: 1.	Motor severity factor: eyes and upper face: 2, lower face: 1, jaw and tongue: 1, larynx: 0, neck: 1, shoulder and proximal arm 0, distal arm and hand (including elbow): 0, pelvis and proximal leg: 0, distal leg and foot (including knee): 0, trunk: 0.
	Total = 22	Total = 5
**2**	Duration factor: 4	Duration factor: 0.5
	Motor severity factor: eyes and upper face: 1, lower face: 4, jaw and tongue: 3, larynx: 0, neck: 4, shoulder and proximal arm. 4, distal arm and hand (including elbow): 4, pelvis and proximal leg: 4, distal leg and foot (including knee): 4, trunk: 1.	Motor severity factor: eyes and upper face: 1, lower face: 0, jaw and tongue: 1, larynx: 1, neck: 1, shoulder and proximal arm. 0, distal arm and hand (including elbow): 0, pelvis and proximal leg: 1, distal leg and foot (including knee): 0, trunk: 0.
	Total = 23	Total = 6.5
**3**	Duration factor: 4	Duration factor: 1
	Motor severity factor: eyes and upper face: 1, lower face: 4, jaw and tongue: 4, larynx: 0, neck: 3, shoulder and proximal arm. 3, distal arm and hand (including elbow): 2, pelvis and proximal leg: 3, distal leg and foot (including knee): 4, trunk: 0.	Motor severity factor: eyes and upper face: 1, lower face: 1, jaw and tongue: 2, larynx: 0, neck: 1, shoulder and proximal arm: 1, distal arm and hand (including elbow): 1, pelvis and proximal leg: 2, distal leg and foot (including knee): 1, trunk: 0.
	Total = 38	Total = 11

## Discussion

Currently, treatment approaches for LN are experimental, as therapeutic targets are not fully elucidated [4,5]. Dysregulation of dopaminergic pathways may be the cause of self-injurious behaviours in LN patients [6] and anatomical/physiological alterations were recently demonstrated in specific brain regions [7]. Impaired dopamine signalling during cerebral development could lead to the compensatory hypersensitivity of dopamine receptors, especially of the D1 subtype: this prevents the success of either dopaminergic drugs (which increase symptoms [8]) and antipsychotics (which do not target D1 receptors). The dopaminergic and GABAergic systems are connected at multiple levels and GABA has a prominent influence on dopamine release in the mesolimbic and nigrostriatal circuits [9]. Moreover, baclofen may serve as a functional antagonist of dopamine: GABA_B_ receptors are coupled to G proteins that inhibit adenylyl cyclase activity, while D1 dopamine receptors activate it. Baclofen may also have a direct anxiolytic effect [10] that could complement its activity on the dopaminergic balance and be useful for behavioural improvement. The use of baclofen and ITB for LN patients is not uncommon, a population study reported on ten users of oral baclofen and one of ITB, although it did not discuss therapeutic efficacy [2]. Good results of ITB therapy were also previously observed in two patients, although only little information was reported [11]. In order to compare available data, debate should be fostered between clinicians with different experiences on baclofen treatment in LN. Our cases further support the use of ITB in patients with LN within a multi-targeted therapy that may ameliorate both motor and behavioural symptoms. ITB may represent a viable therapy for LN patients, especially in light of the severity of this disease and of the lower comparative risk of severe side effects. Nevertheless, catheters for ITB administration may become infected, leading to removal in spite of partial ITB efficacy [12]. The risks of infection may be avoided by oral administration of baclofen, which is in general safe, apart from rare cases of hepatic toxicity [13]. We conclude that baclofen is potentially useful as a therapy for LN, but that additional studies should be conducted, in order to properly assess its efficacy. Both intrathecal and oral administration routes should be investigated, with systematic measurements and long follow-up periods.

## Abbreviations

LN: Lesch-Nyhan syndrome
ITB: Intrathecal baclofen
